# The Mediating Relation between Symbolic and Nonsymbolic Foundations of Math Competence

**DOI:** 10.1371/journal.pone.0148981

**Published:** 2016-02-09

**Authors:** Gavin R. Price, Lynn S. Fuchs

**Affiliations:** 1 Department of Psychology & Human Development, Peabody College, Vanderbilt University, 230 Appleton Place, Nashville, TN, 37203, United States of America; 2 Department of Special Education, Peabody College, Vanderbilt University, 230 Appleton Place, Nashville, TN, 37203, United States of America; University of Leuven, BELGIUM

## Abstract

This study investigated the relation between symbolic and nonsymbolic magnitude processing abilities with 2 standardized measures of math competence (WRAT Arithmetic and KeyMath Numeration) in 150 3^rd^- grade children (mean age 9.01 years). Participants compared sets of dots and pairs of Arabic digits with numerosities 1–9 for relative numerical magnitude. In line with previous studies, performance on both symbolic and nonsymbolic magnitude processing was related to math ability. Performance metrics combining reaction and accuracy, as well as weber fractions, were entered into mediation models with standardized math test scores. Results showed that symbolic magnitude processing ability fully mediates the relation between nonsymbolic magnitude processing and math ability, regardless of the performance metric or standardized test.

## Introduction

The development of effective numerical and mathematical skills is of critical importance for success in today’s society. However, as many as one in four economically active individuals fail to develop those skills [1], placing them at higher risk of unemployment, physical and mental illness, and arrest and incarceration [2,3]. Furthermore, the United States lags behinds its economic peers in terms of math skills development, ranked 21^st^ of 23 countries in a recent report by the Institute of Education Sciences [4]. Due to the cumulative nature of math skills, improving the earliest foundations of math skills offers the best hope for mitigating the burgeoning problem of low numeracy. Therefore a deeper understanding of those foundations must be reached.

A growing body of evidence suggests one critical foundation for math development is the ability to represent and manipulate nonsymbolic numerical magnitude (i.e., the number of items in a set of objects). Studies suggest this ability is present in human infants [5,6], preliterate human cultures [7], and even non-human primates [8,9]. This makes nonsymbolic magnitude processing an ideal candidate mechanism upon which numerical and mathematical skills may be scaffolded.

Nonsymbolic magnitude processing ability varies between individuals and is typically indexed by performance on nonsymbolic numerical comparison tasks, in which individuals judge which of two sets of dots is the more numerous. Performance on this task has been found to correlate with math competence in children and adults [10–12] and is atypical in children with mathematical learning disabilities (dyscalculia) [13]. Furthermore, the brain systems thought to support nonsymbolic magnitude processing are structurally and functionally atypical in children with dyscalculia [14,15]. Such findings lend support to the ‘magnitude representation hypothesis’ that nonsymbolic magnitude processing is a critical foundation for math competence.

Despite the intuitive appeal of the magnitude representation hypothesis, recent evidence suggests that the relation between nonsymbolic magnitude processing and math competence may not be entirely straightforward. A number of studies have reported no significant correlation between nonsymbolic magnitude comparison performance and math competence [16–18]. Instead findings indicate that performance on symbolic number comparison (i.e., comparing the relative magnitude of two Arabic digits) predicts math ability [19–22]. Generally speaking, symbolic number knowledge is well known to predict later math achievement [23,24]. Furthermore, a recent meta-analysis suggests that the relation between symbolic number skills and math is stronger than that between nonsymbolic magnitude processing and math [25].

Such findings suggest first that the relation between nonsymbolic magnitude processing and math competence may not be as robust as initially thought, and second that the ability to represent and process quantity information through symbols (i.e., Arabic digits) may in fact be of greater importance for the development of math skills. This perspective is often referred to as the ‘symbol mapping hypothesis.’ In a recent review,[26] noted that the relation between symbolic number comparison and math achievement appears to be more robust than the relation between nonsymbolic number comparison and math achievement, noting that no clear explanatory pattern is currently evident. It should be noted that two recent meta-analyses identified small but robust relations between nonsymbolic magnitude processing and math achievement [27,28]. Chen and Li (2014) in particular suggest that the failure to find a significant association between nonsymbolic skills and math achievement in some studies may be due to insufficient power to detect what is essentially a small but true relation. Thus, neither nonsymbolic nor symbolic skills can be discounted as foundational competencies for math achievement despite inconsistent findings.

The extant literature provides a conflicting mixture of results, against which a purely nonsymbolic or symbolic account of the foundations of math competence seems insufficient. This suggests the importance of forging a mechanistic account that incorporates both symbolic and nonsymbolic abilities. One approach to solving this puzzle is to consider the developmental trajectory of numerical and mathematical skills. Nonsymbolic skills are either acquired very early in infancy or are innate; basic symbolic number skills are acquired in the years preceding school entry; and basic arithmetic skills are acquired and refined in the first years of elementary school. Such a developmental framework negates the need to position nonsymbolic and symbolic skills as alternative foundations.

Instead, it allows for the hypothesis that nonsymbolic skills influence the acquisition of symbolic number skills, which in turn influence the acquisition of arithmetic skills. In other words, it is possible that symbolic skills *mediate* the relation between nonsymbolic skills and math competence. This ‘mediation hypothesis’ is supported by [29] who showed that numerical ordering skill mediates the relation between nonsymbolic magnitude processing and arithmetic skill in adults, while controlling for performance on symbolic magnitude comparison. These authors suggest that “the representation of order in numerical symbols serves as an ideal stepping stone” (p. 260) between nonsymbolic magnitude processing and math. A second recent study showed that nonsymbolic magnitude processing at 3 years of age predicts math skills measured at 4 years, but that this relation is fully mediated by symbolic number skills measured at 3 and a half years [30]. Those authors suggest that nonsymbolic magnitude processing ability influences math achievement “by facilitating the learning of Arabic numerals, number words and their understanding of the meaning of these symbols” (p. 503). In contrast, another recent study, which tested the relation between nonsymbolic and symbolic number skills and math achievement in fifth graders, did not find evidence for a mediating relation [28]. Instead, that study found evidence for independent contributions of both symbolic and nonsymbolic skills to math ability, albeit a stronger relation between math and symbolic versus nonsymbolic number skills. Thus, the limited extant literature provides an inconsistent account of the mediating role of symbolic number skills on the relation between nonsymbolic magnitude processing acuity and math achievement.

However, closer inspection reveals a pattern that may explain the apparent inconsistencies. The mediation model reported in by vanMarle et al. (2014) considered four possible mediating variables: verbal counting, give-a-number (counting out a requested number of objects from a pile), numeral recognition, and numeral comparison. Results indicate that only verbal counting and give-a-number tasks mediated the relation between nonsymbolic magnitude processing and math achievement. Numeral recognition did not correlate with nonsymbolic magnitude processing, while numeral comparison did not predict math achievement. Thus, the two tasks that did mediate the nonsymbolic magnitude processing—math relation were strongly ordinal in nature, suggesting in concert with Lyons and Beilock (2011) that knowledge of the ordinal relationships between numbers, not necessarily their magnitudes, is the active mediator.

On the other hand, Fazio et al (2014) investigated the mediation relation using tests of magnitude processing as opposed to order processing. The absence of a significant mediation model in their study suggests, therefore, that symbolic *ordering* skills mediate the relations between nonsymbolic magnitude processing and math achievement, while symbolic *magnitude* processing does not. Even so, it is important to note that Fazio et al.’s symbolic ‘magnitude understanding’ variable comprised performance on magnitude comparison of fractions and number line estimation with fractions and whole numbers, while the nonsymbolic magnitude understanding variable comprised performance on magnitude comparison of whole numbers and fractions. Thus, these two variables may capture potentially non-overlapping cognitive dimensions (e.g., visuospatial demands of number line estimation vs. whole number magnitude comparison). Such a lack of overlap at the cognitive level is supported by the fact that the variables did not correlate with one another. The extent of cognitive disparity between these two variables may explain why no mediation relation was observed in that study.

In summary, evidence for the relation between nonsymbolic and symbolic number processing and math achievement is mixed and sometimes contradictory. Two studies suggest this confusion may be resolved by considering symbolic number skills as a mediator between nonsymbolic magnitude processing and math achievement. The results of Lyons and Beilock (2011) and vanMarle et al (2014) suggest that understanding of the ordinal properties of symbolic numbers serves this function. In contrast, Fazio et al. (2014) reported no evidence for a mediating relation between nonsymbolic and symbolic number skills and math achievement. Several questions therefore remain regarding (a) the robustness of the mediating role of symbolic number skills and (b) the cognitive mechanisms underlying any such relation.

The purpose of the present study was to address these issues by testing the hypothesis that symbolic *magnitude* processing mediates the relation between nonsymbolic magnitude processing and math achievement. It is the first study to do so using single, overlapping tasks to index both nonsymbolic and symbolic magnitude processing. Furthermore, we tested our mediation model on two standardized metrics of math achievement: one focused on arithmetic and calculation skills and the other focused on knowledge of the base 10 system and other basic number knowledge. In so doing, we tested the hypothesis that symbolic magnitude processing mediates the relation between nonsymbolic magnitude processing and math ability in the early school years, and that this relation holds true for multiple math achievement indices.

It is important to note that although the major focus in this study is nonsymbolic and symbolic magnitude processing, these are not the only cognitive variables thought to influence the development of math ability. The role of working memory in particular has received much attention in previous research, and working memory capacity has been related to a range of numerical and mathematical abilities (for a review see [31]. Brain regions subserving working memory and numerical magnitude processing show some degree of overlap, notably in the intraparietal sulcus [32], and thus it is possible that working memory may play an influential role in the relation between magnitude processing and math ability. Therefore, in addition to examining the mediation hypothesis described above, we also examined the role of working memory in that relationship.

## Method

### Participants

This study was approved by the Behavioral and Social Sciences IRB Committee at Vanderbilt University. Written assent obtained from all participants, and written consent was obtained from parents/guardians. The sample was drawn from the final cohort of a larger, longitudinal study spanning 4 years [33]. The symbolic and nonsymbolic magnitude comparison tasks were administered only in the final year of the study, and for that reason the current results focus exclusively on 3^rd^ grade children. Participants were recruited from a large metropolitan school district in the southeast United States. We excluded students whose teachers identified them as not speaking English or scored below a standard score of 80 on both subtests of the two-subtest Wechsler Abbreviated Intelligence Scale (T-Scores are reported in Table 1) (WASI; Wechsler, 1999). Our sample consisted of 160 children in 3^rd^ grade, seven of whom had accuracy of 50% on either the symbolic or the nonsymbolic number comparison and were consequently removed from further analysis due to the inability of the weber fraction calculation to fit a model to their data. An additional three were removed due to 100% accuracy on the symbolic task, again yielding no fit for the weber fraction model. Thus, the final sample consisted of 150 children (mean age 9.01 yrs, St Dev = 0.37; 87 Females).

**Table 1:** Descriptive Statistics for Experimental and Cognitive Measures.

Measure		Mean	St Dev
**Symbolic**	Accuracy (% Correct)	87.95	8.31
	Reaction Time (ms)	747.61	120.55
	P	926.33	222.08
	*W*	.33	.23
**Nonsymbolic**	Accuracy (% Correct)	81.7	7.94
	Reaction Time (ms)	728.89	106.19
	P	993.17	202.62
	*W*	.48	.27
**WRAT Arithmetic**	Standard Scores	99.54	15.8
**KeyMath Numeration**	Standard Scores	102.42	11.8
**WRAT Reading**	Standard Scores	99.33	14.58
**Working Memory Test Battery Mazes Recall**	Standard Scores	4.03	3.7
**Working Memory Test Battery Word List Recall**	Standard Scores	15.00	3.66

### Experimental Tasks and Cognitive Measures

#### Nonsymbolic Number Comparison

Participants were simultaneously presented two sets of dots and asked to indicate via button press which set was more numerous (i.e., which set contained more dots). Stimuli were presented for 1000ms and separated by 1000ms fixation. Dot sets ranged in value from 1–9. 48 number pairs were presented, with the ratio between sets (ratio = smaller number/larger number) ranging from 0.2–0.777. Numbers pairs were assigned to one of three ratio ‘bins’: small, medium, or large. The small ratio condition contained 16 trials with ratios of 0.2, 0.222, and 0.25. The medium ratio condition contained 16 trials with ratios of 0.5 and 0.556. The large ratio condition contained 16 trials with ratios of 0.714, 0.75, and 0.778. Split-half reliability estimates for this task have been reported range from .47 to .88 [17,34], and test-retest reliability from .06 to .79 [35]. Cronbach’s alpha’s for accuracy and reaction time in this sample were .87 and .94 respectively.

To control for the possibility that visual cues drive task results rather than judgment of numerosity, we randomly varied four visual parameters using the method presented in Gebuis and Reynvoet (2014). Total surface area of dots, area extended by dots, average dot diameter, and dot density were manipulated to provide two sets of dots for each trial where visual cues indicated a larger numerosity (congruent) or were contraindicative (incongruent) in approximately half of the total trials. Regression analyses predicting the number ratio of dot sets (larger–smaller/ larger) from stimulus parameter (difference in visual properties) confirmed that no single visual parameter significantly predicted number ratio.

#### Symbolic Number Comparison

Participants were shown two simultaneously presented Arabic digits and asked to indicate via button press which of the two was numerically larger (e.g., 8 is larger than 3). The values of the digits and number pairs were identical to those for the nonsymbolic comparison task. Cronbach’s alpha’s for accuracy and reaction time in this sample were .80 and .97 respectively.

#### Achievement Measures

Arithmetic: *The WRAT Arithmetic subtest* includes oral and written items including counting, reading number symbols, solving oral problems and written computations ranging from simple arithmetic to calculus. At this age, alpha has been reported at .87 (Fuchs et al., 2014). The manual reports split-half reliability of .94.

Numeration: *The KeyMath Numeration subtest* measures early number awareness including place value and number sense, magnitude of numbers, fractions, decimals, percentages, exponents, integers, multiples, and factors. Reliability for this measure has been reported as in “the upper .80s and 90s” [36].

Reading: *The WRAT Reading Subtest* requires participants to read aloud letters and words until ceiling is reached. The manual reports split-half reliabilities of .98.

Working Memory: *Working Memory Test Battery for Children*, *Mazes Recall* (Pickering & Gathercole, 2001). This test requires participants to recall routes drawn through two-dimensional mazes of increasing complexity. *Working Memory Test Battery for Children*, *Word List Recall* (Pickering & Gathercole, 2001). This test requires participants to recall a spoken list of monosyllabic words.

#### Experimental Task Metrics

To analyze performance on the nonsymbolic and symbolic number comparison tasks, we computed two performance metrics, one to index overall performance and one to index the impact of ratio on performance. The first metric, ‘*Performance’* (P), was based on the method reported by [37] and combines reaction time and error rates according to the following formula; P = RT(1+2ER). P is conceptually similar to the commonly used measure inverse efficiency (RT/Accuracy) in that it essentially corrects reaction times according to error rates and controls for speed-accuracy trade-offs. The second metric we computed was ‘*weber fraction’(w)*. *w* derives from the Weber-Fechner Law and is essentially a metric of the noise in an individual’s representation of numerical magnitude. *w* can be thought of as indexing the ratio between two numbers necessary to consistently discriminate those numbers with 75% accuracy and is therefore a metric of the individuals sensitivity to the ratio between the two numbers being compared. To compute our *w* scores we used the formula employed by[38]. Kolmogorov-Smirnoff tests indicated that P was not normally distributed for either nonsymbolic (*p* < .001) or symbolic (*p* = .010) comparison. The same was true for nonsymbolic (*p* < .001) and symbolic (*p* < .001) weber fractions. Therefore we log transformed each of the variables. The distributions of log10 values for symbolic and nonsymbolic P and nonsymbolic *w* were normal (*p* > .05 for each), while symbolic *w* remained skewed (*p* = .002). However, the transformation did reduce the skewness of symbolic *w* from 1.845 to 0.347, indicated an improved distribution. Therefore the log transformed values for each variable were used for subsequent analyses. Outlier correction was carried out to remove trials which fell outside 3 standard deviations above or below the participant’s mean reaction time.

## Results

The means and standard deviations for the experimental (Symbolic P and *w*, nonsymbolic P and *w*) and cognitive measures are reported in Table 1 (data in S1 Table). Note that the w scores observed in the current sample are higher than those reported in some previous studies using samples of children of similar ages. The reason for this discrepancy is not clear; however, differences in overall accuracy, as well as the distribution of accuracies across ratios between studies may influence estimations of individual weber fractions.

### Correlations

First, we replicated previous findings by showing significant correlations between each of our experimental metrics and math achievement. Pearson’s *r* coefficients among our experimental metrics and achievement measures are reported in Table 2. Because our nonsymbolic comparison paradigm included quantities in the subitizable range, we computed the above correlations after separating the nonsymbolic trials into those with numerosities of less than or equal to 4 (subitizable) and with numerosities above 4 (nonsubitizable). Splitting the trials in this manner reduced trial numbers to the extent that we were unable to fit reliable models for *w* scores; so, this analysis reports P scores only. Furthermore, to account for possible influences of ordering ability in the symbolic comparison task, we split symbolic comparison trials into ascending (i.e., right number larger than left) and descending pairs (i.e., left number larger than right). Again, this reduced the number of trials to the extent that we were not able to fit reliable models for *w* scores; so, this analysis reports P scores only.

**Table 2:** Correlations between experimental and cognitive measures.

	1	2	3	4	5	6	7	8	9	10	11	12
**1. WRAT Arithmetic (Standard Score)**												
**2. KeyMath Numeration (Standard Score)**	.65**											
**3. Symbolic P (Log10)**	-.46**	-.52**										
**4. Symbolic P Ascending (Log10)**	-.44**	-.49**	.96**									
**5. Symbolic P Descending (Log10)**	-.47**	-.51**	.95**	.83**								
**6. Symbolic *w* (Log10)**	-.40**	-.40**	.74**	.73**	.67**							
**7. Nonsymbolic P (Log10)**	-.30**	-.43**	.71**	.70**	.66**	.37**						
**8. Subitizable Nonsymbolic P (Log10)**	-.33**	-.41**	.67**	.41**	.41**	.34**	.97**					
**9. Nonsubitizable Nonsymbolic P (Log10)**	-.28**	-.42**	.71**	.44**	.44**	.40**	.98**	.96**				
**10. Nonsymbolic *w* (Log10)**	-.29**	-.36**	.52**	.51**	.47**	.51**	.71**	.70**	.71**			
**11. WRAT Reading (Standard Score)**	.50**	.60**	-.25*	-.24**	-.23**	-.18*	-.29**	-.31**	-.29**	-.22**		
**12. Mazes Recall (Raw Score)**	.28**	.29**	-.30**	-.27*	-.30*	-.24*	-.19**	-.17*	-.17*	-.18*	.22**	
**13. Word List Recall (Raw Score)**	.25**	.27**	-.19*	-.16*	-.20*	-.19*	-.24**	-.23**	-.26**	-.24**	.33**	.21**

** p < .01

*p < .05

### Mediation Analyses

To investigate the potential mediating role of symbolic number comparison in the relation between nonsymbolic comparison and math achievement, we conducted four mediation analyses using SPSS v20 and Preacher and Hayes’s (2008) ‘INDIRECT’ macro. The indirect effect was examined by the generation of confidence intervals with the endpoints determined by the bias-corrected and accelerated method (Hayes, 2009). If zero is outside the confidence interval, the indirect effect is consequently not zero (Hayes, 2009). WRAT Reading standard scores, mazes recall, and word list recall were included as covariates in these analyses to control for reading ability, visuospatial and phonological working memory respectively.

Note that mediation analyses are correlational; so causation should not be inferred. This is especially true in the present study because the independent, mediating, and dependent variables were collected concurrently. That said, the variables used in the present study can be assumed to measure skills acquired in the appropriate chronological order. A large body of research indicates that nonsymbolic magnitude processing skills are evident within the first months of infancy[39–41]. Arabic digits are typically learned in the years prior to school entry [42,43], and formal math skills are typically learned during the first years of school [44].

**Fig 1:**
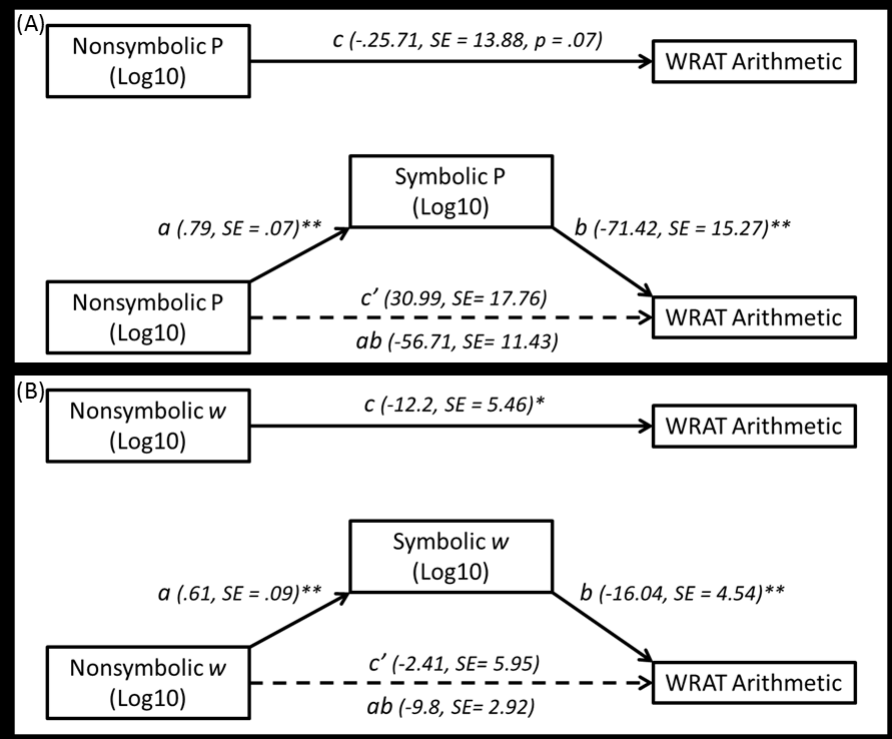
Mediation models for WRAT Arithmetic using (A) log transformed P scores and (B) log transformed *w* scores.

**Fig 2:**
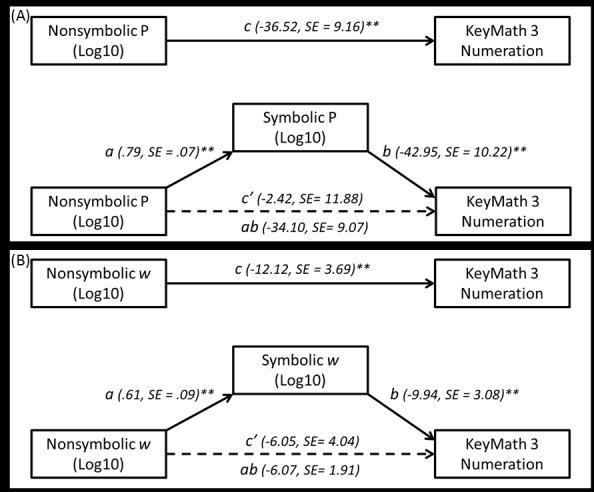
Mediation models for KeyMath 3 Numeration using (A) log transformed P scores and (B) log transformed *w* scores.

The coefficients and standard errors for the mediation models using WRAT Arithmetic as the dependent variable. As shown, the indirect effect (ab path) was significant for each analysis, while the c’ path was not, and importantly, the confidence intervals did not contain zero for any of the models (Table 3). These results indicate full mediation whereby the relation between math skills by nonsymbolic number processing was completely accounted for by symbolic number processing skills (Figs 1 and 2).

**Table 3:** Confidence intervals for all mediation models.

Independent Variable	Mediator	Dependent Variable	Lower CI	Upper CI
Nonsymbolic P	Symbolic P	WRAT Arithmetic	-81.25	-34.61
Nonsymbolic w	Symbolic w	WRAT Arithmetic	-15.95	-4.61
Nonsymbolic P	Symbolic P	KM Numeration	-53.56	-18.39
Nonsymbolic w	Symbolic w	KM Numeration	-10.99	-3.11
Symbolic P	Nonsymbolic P	WRAT Arithmetic	-8.02	39.3
Symbolic w	Nonsymbolic w	WRAT Arithmetic	-5.43	3.8
Symbolic P	Nonsymbolic P	KM Numeration	-15.87	13.09
Symbolic w	Nonsymbolic w	KM Numeration	-5.78	0.74

To account for possible influences of the nonsymbolic task including sets in subitizing range, we replicated the mediation models after separating the nonsymbolic trials into those with numerosities of less than or equal to 4 (subitizable) and below and those with numerosities above 4 (nonsubitizable). Splitting the trials in this manner reduced trial numbers to the extent that we were unable to fit reliable models for *w* scores; so, this analysis reports P scores only. As with the main analyses, all models showed significant indirect effects and confidence intervals did not include zero, indicating full mediation. Furthermore, to account for possible influences of ordering ability in the symbolic comparison task, we split symbolic comparison trials into ascending (i.e., right number larger than left) and descending pairs (i.e., left number larger than right). Again, this reduced the number of trials to the extent that we were not able to fit reliable models for *w* scores; so, this analysis reports P scores only. As with the main analyses, all models showed significant indirect effects and confidence intervals did not include zero, indicating full mediation.

To investigate the extent to which working memory ability mediated the relation between nonsymbolic magnitude processing and math ability, we replicated the four mediation models above, but instead of including our two working memory measures as covariates we included them as possible mediators in the model. In all four models, the mediating role of symbolic magnitude comparison remained significant, while for working mazes memory and word list recall scores, the direct effects on math (b paths) were not significant (p > .05 in all cases). Furthermore, in contrast to symbolic magnitude processing, for both working memory measures, the bias corrected confidence intervals for the test of indirect effects contained zero, indicating a lack of mediation. Therefore it appears that the relation between nonsymbolic magnitude processing and math ability is independent of working memory capacity.

It seems intuitively logical that nonsymbolic magnitude processing abilities influence the acquisition of symbolic number skills, because nonsymbolic magnitude processing abilities are evident in early infancy, while symbolic skills are not. However, a range of evidence suggests that this relation may be bidirectional, with the acquisition of symbolic skills prompting a refinement of nonsymbolic magnitude representations [45]. Given that the current sample comprised 3^rd^-grade children who were highly familiar with Arabic digits, it is possible that by this age, symbolic number skills had exerted and influence on nonsymbolic magnitude representations. Therefore, we replicated the above mediation analyses, but entered symbolic magnitude processing as the independent variable and nonsymbolic magnitude processing as the mediator. In all four models the confidence intervals contained zero (Table 3), suggesting that nonsymbolic magnitude processing does not mediate the relation between symbolic magnitude processing and math performance.

## Discussion

A growing body of literature suggests that the ability to compare numerical magnitudes serves a critical foundation for math development. Some studies have shown that nonsymbolic magnitude processing predicts math ability, while others have shown that symbolic magnitude processing is instead the key predictor (for a review see [26]). This conflicting pattern of results has led to confusion as to how symbolic and nonsymbolic number skills respectively scaffold later math abilities. An alternative hypothesis considers a developmental framework whereby nonsymbolic skills influence later math ability by facilitating the acquisition of symbolic skills. The present study provides evidence in support of this hypothesis, showing that symbolic magnitude comparison fully mediates the relation between nonsymbolic magnitude comparison and math ability on two separate math achievement measures, even when controlling for reading ability and working memory.

The present findings address an emerging debate regarding this ‘mediation hypothesis’ and the role symbolic number knowledge plays in facilitating the relation between nonsymbolic number processing and math achievement. Lyons and Beilock (2011) found that numeral ordering ability mediates the relation between nonsymbolic magnitude processing and math achievement in adults, when controlling for symbolic magnitude comparison. Those results left open the question of whether symbolic *magnitude processing*, in addition to ordering ability, mediates the relation between nonsymbolic magnitude processing and math ability. Subsequently, vanMarle et al. (2014) reported that symbolic number knowledge mediates the relation between nonsymbolic magnitude processing in preschoolers. However, the mediating relation reported in that study was driven by the effects of counting based tasks, namely, verbal counting and ‘give-a-number,’ which essentially tap into ordering as opposed to magnitude processing abilities. Thus, the question of whether basic symbolic *magnitude* processing mediates the relation between nonsymbolic magnitude processing and math achievement remains open.

Recently reported data from Fazio et al. (2014) used magnitude processing tasks, including number line estimation, fraction comparison and whole number comparison, to investigate the mediation hypothesis. Their results revealed no evidence of a mediating relation, but instead independent contributions of symbolic and nonsymbolic number processing to math achievement. However, that study’s metrics of symbolic and nonsymbolic magnitude processing were cognitively dissimilar due to their composition, thus complicating the interpretation of the absence of mediating relation.

The present results provide evidence that is convergent and consistent with several previous studies showing that symbolic number skills mediate the relation between nonsymbolic magnitude processing and math ability. While previous studies have provided evidence for this mediating relation using measures primarily engaging symbolic ordering processes, the present study shows that basic symbolic *magnitude* processing also mediates the relation between nonsymbolic magnitude processing and math ability. Two further points are also noteworthy. First, the mediation models were significant for both our measure of general task performance (P) as well as our measure of ratio sensitivity (*w)*. While *w* is widely considered to be a metric of the acuity of the mental representation of numerical magnitude, several recent studies have called into question the extent to which it represents a superior metric to mean accuracy [34,35] or other more general task performance metrics. The present data suggest no significant advantage of using *w* over P.

The second noteworthy point is that the mediation models were significant for both our measures of math achievement. Our first measure was the Arithmetic subtest of the WRAT, which includes oral and written items including counting, reading number symbols, solving oral problems and written computations ranging from simple arithmetic to calculus. The second measure was the Numeration subtest of the KeyMath3 battery, which measures early number awareness including place value and number sense, magnitude of numbers, fractions, decimals, percentages, exponents, integers, multiples, and factors. While some overlap in these measures exists, the Numeration test can be thought of as measuring more basic numerical knowledge, while the WRAT Arithmetic subtest measures acquired formal calculation skills.

The fact that our mediation model was significant for both measures suggests that the developmental mediation framework is a general one that relates to a broad range of mathematical and numerical abilities. It is interesting to note, however, that although both models were significant, the direct effect of nonsymbolic magnitude processing was marginally nonsignificant (p = .07) in the mediation model for the performance (P) variable. This effect was significant when word list recall was not included in the model, suggesting perhaps that WRAT arithmetic is more influenced by phonological working memory than KeyMath Numeration. This suggests that the two measures of math ability are partially but not completely overlapping. It also suggests that while symbolic magnitude processing seems to facilitate development of a wide range of math skills, nonsymbolic magnitude processing may have a stronger relation with some acquired math skills than others. This variation is of relevance for the development of educational interventions that seek to improve math skills by enhancing Nonsymbolic magnitude processing. Improving that acuity may be of greater benefit for basic number skills than for formal arithmetic.

The present study also investigated the role of working memory in the relation between nonsymbolic magnitude processing and math ability. Our results indicated via bivariate correlations that working memory measures were associated with math outcomes. However, mediation models indicated that this relation did not influence the relation between nonsymbolic magnitude processing and math outcomes. These results suggest, therefore, that while both nonsymbolic magnitude processing and working memory are related to math achievement, those relations are independent of one another.

It is important to acknowledge that because the present data were collected concurrently, the ability to draw inferences regarding the causal pathways between our independent variables is limited, as it is possible that the acquisition of symbolic number knowledge has a reciprocal influence on nonsymbolic magnitude processing skills [45,46]. At the same time, the mediation model we tested is in line with a theoretical perspective with strong grounding in existing evidence. Nonsymbolic magnitude processing skills are observable in human infants long before symbolic skills are acquired, and nonsymbolic skills have been shown to predict later math ability in longitudinal research (e.g. [11]). However, we tested the alternate hypothesis that nonsymbolic magnitude processing mediated the relation between symbolic magnitude processing and math performance by reversing the symbolic and nonsymbolic magnitude processing in the model. In each alternate model, results suggested that symbolic magnitude processing had not yet exerted an influence on nonsymbolic magnitude processing. Thus, while the present results suggest that nonsymbolic magnitude processing influences symbolic processing and not the other way around, it is possible that the alternate pathway emerges later in development. Future longitudinal research that collects measures at sequential time points and across different age groups is required to fully disentangle this open empirical question.

We also note that while the symbolic task used is a simple magnitude comparison judgment, some processing of numerical order may influence performance on this task. Given that participants always had to select the larger of the two numbers and that the position of the larger number on the left or the right was counterbalanced, it seems unlikely that ordering would be a uniform strategy across the entire task, but ordering strategies may have been invoked in a subset of trials. Thus, future studies should include explicit ordering tasks to comprehensively disentangle the contributions of magnitude processing and order processing to the proposed mediation framework.

Furthermore, it should be noted that much of the recent literature refers to nonsymbolic magnitude processing in the context of an ‘Approximate Number System’ (ANS), a cognitive system that allows the ordering and comparison of magnitudes without use of verbal or visual labels. Given that the current study employed a nonsymbolic comparison task that included dots within the subitizing range, we cannot be certain that *only* the ANS was engaged in the resolution of that task, and therefore we refer to nonsymbolic magnitude comparison and not ‘ANS acuity’. Future studies should seek to replicate the current findings using only numerosities outside the subitizing range.

A final limitation of the present study is that our model did not control for inhibitory control processes. Some recent studies have suggested that performance on nonsymbolic comparison tasks may be driven, at least in part, by individual differences in inhibitory control processes [18,47]. Thus future research should control specifically for inhibitory control when assessing the mediating role of symbolic magnitude processing so as to more completely characterize what is being mediated.

In conclusion, results of the present study are consistent with the theory that Nonsymbolic magnitude processing influences math ability by facilitating the learning of Arabic digits. These results are also consistent with results from previous studies showing that symbolic numerical ordering ability mediates the relation between nonsymbolic magnitude processing and math ability. Thus, the mediating role of symbolic number skills appears to encompass both magnitude and ordering abilities. A more precise representation of numerical magnitude allows for more effective mapping of Arabic digits onto mental representations of numerical magnitude. This stronger mapping subsequently facilitates more effective acquisition of formal mathematical skills. Further research is required to establish more precisely the interplay between ordinal knowledge versus magnitude knowledge and their respective contributions to this mediating framework.

## Supporting Information

S1 Table Study data used for reported analyses. (SAV)
